# DHA modulates MANF and TREM2 abundance, enhances neurogenesis, reduces infarct size, and improves neurological function after experimental ischemic stroke

**DOI:** 10.1111/cns.13444

**Published:** 2020-08-05

**Authors:** Ludmila Belayev, Sung‐Ha Hong, Raul S. Freitas, Hemant Menghani, Shawn J. Marcell, Larissa Khoutorova, Pranab K. Mukherjee, Madigan M. Reid, Reinaldo B. Oria, Nicolas G. Bazan

**Affiliations:** ^1^ Neuroscience Center of Excellence, School of Medicine Louisiana State University Health New Orleans New Orleans LA USA; ^2^ Present address: UT Health University of Texas Health Sciences Center at Houston McGovern Medical School Houston TX USA; ^3^ Present address: Laboratory of the Biology of Tissue Healing, Ontogeny and Nutrition Department of Morphology and Institute of Biomedicine School of Medicine Federal University of Ceara Fortaleza Brazil

**Keywords:** astrocytes, ischemic stroke, MANF, microglia, neurogenesis, neuroprotection, TREM2

## Abstract

**Aims:**

Mesencephalic astrocyte‐derived neurotrophic factor (MANF) is a secretory neurotrophic factor protein that promotes repair after neuronal injury. The microglia cell surface receptor (triggering receptor expressed on myeloid cells‐2; TREM2) regulates the production of pro‐ and antiinflammatory mediators after stroke. Here, we study MANF and TREM2 expression after middle cerebral artery occlusion (MCAo) and explore if docosahexaenoic acid (DHA) treatment exerts a potentiating effect.

**Methods:**

We used 2 hours of the MCAo model in rats and intravenously administered DHA or vehicle at 3 hours after the onset of MCAo. Neurobehavioral assessment was performed on days 1, 3, 7, and 14; MANF and TREM2 expression was measured by immunohistochemistry and Western blotting.

**Results:**

MANF was upregulated in neurons and astrocytes on days 1, 7, and 14, and TREM2 was expressed on macrophages in the ischemic penumbra and dentate gyrus (DG) on days 7 and 14. DHA improved neurobehavioral recovery, attenuated infarct size on days 7 and 14, increased MANF and decreased TREM2 expression in ischemic core, penumbra, DG, and enhanced neurogenesis on Day 14.

**Conclusion:**

MANF and TREM2 protein abundance is robustly increased after MCAo, and DHA treatment potentiated MANF abundance, decreased TREM2 expression, improved neurobehavioral recovery, reduced infarction, and provided enhanced neuroprotection.

## Graphical abstract

MANF and TREM2 were upregulated following focal cerebral ischemia. DHA treatment enhanced MANF abundance in neurons and astrocytes and suppressed TREM2 expression in activated microglia and astrocytes, which contributed to neurogenesis and neurological recovery.

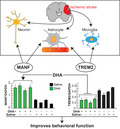

## 1 INTRODUCTION

Cerebral ischemia activates endogenous reparative processes, such as the increased proliferation of neural stem cells (NSCs) in the subventricular zone (SVZ) and migration of neural progenitor cells (NPCs) toward the ischemic area. However, most NPCs die shortly after ischemia and are unable to arrive at the infarct area, limiting this reparative process. Mesencephalic astrocyte‐derived neurotrophic factor (MANF)^1^ belongs to the family of evolutionally conserved neurotrophic factors. It is a 20 kDa secreted protein expressed in glia, neurons, and other cells. MANF targets midbrain dopaminergic neurons,^1^ is neuronal protective, and regulates NPC migration.^2^, ^3^, ^4^ MANF expression and protein abundance are increased in the cerebral cortex after brain ischemia.^5^ The significance and cellular location of MANF in ischemic stroke could also be important to investigate where the inflammatory response is extensive and contribute to the speed of recovery after stroke.^6^

Focal cerebral ischemia initiates a cascade of molecular, cellular, and metabolic events that lead to irreparable brain damage.^7^ Clearing of cellular debris by macrophages after brain ischemia is an important mechanism to sustain homeostasis and promote functional recovery.^8^ Microglial activation is a neural defense against damage triggered by brain ischemia.^9^ Ischemic stroke perturbs endogenous inhibitory signaling and triggers microglial activation^10^ that either aggravates ischemic injury or induces repair and regeneration, depending on the different signals received by microglial receptors.^10^, ^11^ TREM2 abundance on the microglia cell surface is 300 times higher than on astrocytes.^12^ TREM2, coupled with the transmembrane signaling adaptor DAP12, is involved in physiological processes, including proinflammatory responses and phagocytosis of cell debris from damaged neurons and Aβ protein.^13^, ^14^ Alteration of TREM2 expression levels in vitro and in vivo regulates the production of pro‐ and antiinflammatory mediators as well as the conversion of microglial phenotypes after ischemic stroke.^15^ TREM2 exerts antiinflammatory properties by suppressing inflammatory responses along with repression of cytokine production and secretion. The outcome is protection against cerebral ischemia/reperfusion injury through a postischemic inflammatory response and attenuation of neuronal apoptosis.^16^, ^17^ While the activation of postischemic inflammation by the innate immune response is an essential step in the progression of cerebral ischemic injury, the significance of TREM2 in the pathogenesis of ischemic stroke remains to be further elucidated.

At the onset of cerebral ischemia, free arachidonic acid (AA; 20:4, n‐6) and docosahexaenoic acid (DHA; 22:6, n‐3) rapidly accumulate due to increases in intracellular calcium and activation of phospholipases.^18^ Subsequently, a succession of inflammatory mediators, both pro and anti, are generated by enzyme‐mediated free‐radical peroxidation that contributes to cell survival or programmed cell death.^19^ DHA is neuroprotectant against experimental stroke.^20^, ^21^, ^22^ Recently, we demonstrated that DHA decreases infarct volume, improves neurological recovery, and promotes cell survival in the ischemic penumbra as well as the resolution of cerebral edema after focal cerebral ischemia in rats.^20^ The objective of the current study was to define the expression of MANF and TREM2 after focal cerebral ischemia and to investigate whether administration of DHA affects MANF and TREM2 expression and neurogenesis and provides additional neuroprotection.

## 2 METHODS

### 2.1 Animals and surgical preparation

Male Sprague‐Dawley rats (279‐340 g) obtained from Charles River Laboratories were used in all studies. Atropine sulfate (0.5 mg/kg, i.p.) was injected 10 minutes before anesthesia. Anesthesia was induced with 3% isoflurane in a mixture of 70% nitrous oxide and 30% oxygen. All rats were orally intubated and mechanically ventilated. During ventilation, the animals were paralyzed with pancuronium bromide (0.6 mg/kg, i.p.). For blood sampling and infusion of drugs, catheters were placed into the right femoral artery and vein. Serial analyses of arterial blood gases, plasma glucose, hematocrit, and arterial blood pressure were conducted before and during surgical procedures. Rectal (CMA/150 Temperature Controller, CMA/Microdialysis AB) and cranial (temporalis muscle; Omega Engineering) temperatures were maintained at 36°C to 37°C before, during, and after MCAo. Rectal temperature and body weight were monitored daily during the survival period.

### 2.2 Transient middle cerebral artery occlusion (MCAo)

An intraluminal filament coated with poly‐L‐lysine was used to temporarily occlude the right middle cerebral artery for 2 hours, as we previously described.^23^ Four cm of 3‐0 monofilament nylon suture was inserted via the proximal external carotid artery into the internal carotid artery and middle cerebral artery, a distance of 20‐22 mm from the common carotid artery bifurcation. Animals were tested at 60 minutes of MCAo to confirm the presence of a high‐grade neurological deficit. At 2 hours after MCAo, temperature probes were reinserted, intraluminal sutures were carefully removed, and the animals were allowed to survive for 1, 3, 7, or 14 days according to experimental design with free access to food and water.

### 2.3 Assessment of functional neurological outcome

The primary behavioral endpoint was a composite neurological score (0‐12 points, 0 = normal to 12 = maximal deficit).^23^ The battery encompassed to evaluate neurological function: (a) the postural reflex test, to examine upper body posture while the animal is suspended by the tail, and (b) the forelimb placing test, to examine sensorimotor integration in forelimb placing responses to visual, tactile, and proprioceptive stimuli. An observer blinded to the treatment groups performed tests between 9:00 AM and 4:00 PM Tests were performed at 60 minutes (during MCAo) and then on days 1, 2, 3, 7, and 14 after MCAo. The severity of stroke injury was assessed by behavioral examination of each rat, and only animals with a neurological deficit (10 or greater) were used in this study.

### 2.4 Treatments and experimental protocols

Treatment groups included DHA (5 mg/kg, Cayman) or vehicle (0.9% saline) to which rats were randomly assigned. At 3h after the onset of MCAo, an investigator blinded to the treatment groups administered all treatments intravenously using an infusion pump at a constant rate over 3 minutes. Previously, we investigated DHA dose‐response study, which showed that a 5 mg/kg dosage was highly neuroprotective in focal cerebral ischemia^24^; therefore, this dose was utilized in this study. All treatments were administered by researchers blinded to the treatment groups.

**FIGURE 1:**
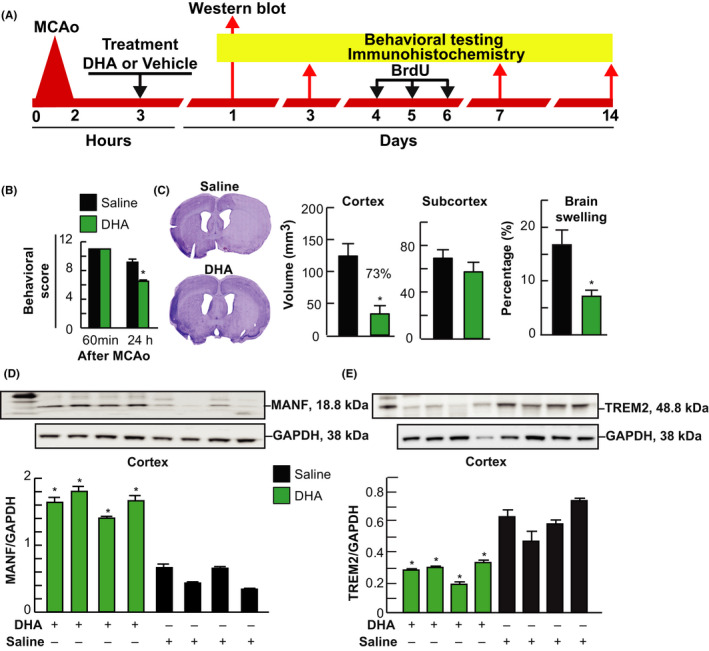
DHA increased MANF expression, decreased TREM2 expression, attenuated infarct size, and led to neurological protection 24 h after MCAo. Rats were treated with DHA or saline at 3 h after onset of MCAo and sacrificed 24 h later. **A**, Experimental design for series 1 and 2; **B**, Total neurological score (normal score = 0, maximal deficit = 12) in DHA and saline‐treated groups; **C**, Computer‐generated MosaiX‐processed images of Nissl‐stained brain sections and cortical, subcortical infarct volume and brain swelling; **D, E**, Western blot analysis of MANF/GAPDH and TREM2/GAPDH expression in ipsilateral cortex and subcortex. Value is mean ± SEM N = 6/group, *P < .05, DHA vs saline, Student *t* test

Two sets of experiments were conducted following the protocols (Figure 1A). In series 1, the effect of DHA on MANF and TREM2 expression during 24 hours survival was evaluated: DHA or saline (6‐8 rats/ group), neurobehavioral testing, histopathology, MANF immunohistochemistry (IHC), and MANF and TREM2 Western blot analysis were performed. In series 2, the effect of DHA on long‐term survival, MANF and TREM2 expression, and neurogenesis: DHA or saline (5‐6 rats/ group), neurobehavioral testing and MANF and TREM2 IHC on days 1, 3, 7, and 14, in vivo labeling with 5‐bromo‐2’‐deoxyuridine (BrdU) on days 4, 5, and 6, and histopathology on Day 14.

### 2.5 5‐Bromo‐2’‐deoxyuridine (BrdU) in vivo labeling

Proliferated cells were followed using the thymidine analog 5‐Bromo‐2ʹ‐deoxyuridine (BrdU). BrdU (50 mg/kg, in saline, Sigma) was administered IP on days 4, 5, and 6 after MCAo.^25^ On day 14, after MCAo rats were perfused with 0.9% saline, followed by 4% paraformaldehyde. Brains were then extracted and subjected to immunohistochemistry. To denature DNA, the sections were incubated in 2 N HCl at room temperature for 1 hour, then neutralized with 0.1 mol/L boric acid (pH 8.5) twice for 5 minutes, and immunohistochemistry was performed as described below.

### 2.6 Histopathology and immunostaining

Animals were allowed to survive for 1, 3, 7, and 14 days, reanesthetized, and transcardially perfused with saline followed by 4% paraformaldehyde; brains were removed and embedded in a gelatin matrix using MultiBrain™ Technology (NeuroScience Associates).^26^ Nissl and ischemia contrast staining (ICS) were used to identify brain infarction. For ICS, sections were first mounted onto gelatinized (subbed) slides, then stained with a modification of the Weil method for myelin.^27^ Unlike myelin staining, the gray matter is left dark in order to highlight ischemic areas. Infarct volume was quantified using digitized histological section (MCID core imaging software; InterFocus Imaging Ltd., Cambridge, England) at nine standardized coronal levels (bregma levels: +5.2, +2.7, +1.2, −0.3, −1.3, −1.8, −3.8, −5.0, and −7.3 mm) using a CCD camera (QICAM Fast 1394, QIMAGING).^23^ Imaging of brain sections was performed on a motorized microscope BX61VS (Olympus) at 10× objective. The zones of infarction (which were clearly demarcated) were then outlined by an investigator blinded to the experimental groups, as well as the left and right hemispheres of each section. To calculate infarct volume, the combined product of the cross‐sectional area and inter‐sectional distance corrected with brain swelling was used.^23^ Brain edema was measured by the differences between ipsilateral and contralateral hemispheres.^23^

Immunohistochemistry was performed on adjacent sections on days 1, 3, 7, and 14 using the following antibodies: TREM2 (1:500; LS Biosciences), Iba1 (1:2000; Wako), GFAP (1:500; Daco), NeuN (1:200; Chemicon Inc), MANF (1:100, Thermo Fisher Scientific), and anti‐BrdU (AbD Serotec), and Hoechst 33342 (Sigma, Oakville, ON, Canada).^28^ The number of positive cells was counted in the dentate gyrus (DG), crest, lateral blade (suprapyramidal portion) and medial blade (infrapyramidal portion), subventricular zone (SVZ; at bregma level −3.8 mm), hippocampus (CA1 and CA3), and at the level of the central lesion in the cortex and striatum (bregma level −0.3 mm). Data were expressed as the quantity of positive cells per high‐power microscopic field (magnification ×40). Fluorescent intensity was analyzed and measured in brain areas.^28^ A confocal laser microscope (LSM510, Carl Zeiss MicroImaging) was used to obtain images of the sections. A dimension of 212.3 µm×212.3 µm was used to acquire the images with Zen software (Carl Zeiss MicroImaging) and ImageJ software (NIH).

### 2.7 Western blot

Western blot analysis was performed at 24 hours after MCAo from the ipsilateral cortex and subcortical regions. Brain protein abundance was determined by Bio‐Rad Assay Method. A total of 20‐25 μg of equivalent protein was loaded on NuPAGE 4%‐12% Bis‐Tris gels and run at 125 V for 120 minutes, and then, proteins were transferred onto PVDF (Invitrogen) paper. TREM2 and MANF proteins were detected using anti‐TREM2 (Millipore‐Sigma) and anti‐MANF antibody (Abcam). Finally, TREM2 and MANF proteins were imaged in Fujifilm LAS‐3000 using Amersham™ ECL™ Western Blotting Detection Reagents. TREM 2 and MNAF protein bands were quantified by Multigage software, and the results were expressed as TREM2/GAPDH and MANF/GAPDH ratio, respectively.

### 2.8 Statistical analysis

Values are reported as means ± SEM. Two‐group comparisons were performed using two‐tailed Student's *t* test. For multiple‐group comparisons, repeated‐measures analysis of variance (ANOVA), followed by Bonferroni tests, was performed. A value of *P* < .05 was regarded as statistically significant.

## 3 RESULTS

### 3.1 Physiological variables

Arterial blood gases, plasma glucose, and rectal and cranial (temporalis muscle) temperatures showed no differences between DHA‐ and saline‐treated groups. Also, there were no adverse side effects and mortality observed in all treatment groups.

### 3.2 DHA improves behavior, attenuates infarct size, upregulates MANF expression, downregulates TREM, and improves during 24h survival

Rats treated with DHA improved the total neurological score by 29% compared to the corresponding vehicle‐treated group at 24 hours after MCAo (Figure 1B). In Figure 1C, representative images of Nissl‐stained brain sections from DHA‐ or saline‐treated rats after MCAo are presented. Saline‐treated showed large infarct over the cortex and subcortex, while DHA‐treated rats demonstrated smaller infarct, mainly located in the subcortex (Figure 1C). DHA treatment dramatically reduced brain swelling as well as cortical infarct volume compared to vehicle‐treated rats (Figure 1C).

Western blot analysis of MANF and TREM2 expression from ipsilateral penumbra region (cortex) is presented in Figure 1D,E and Figure S1‐S2. DHA treatment increased MANF/GAPDH and decreased TREM2/GAPDH expression in cortex 24 hours after MCAo.

**FIGURE 2:**
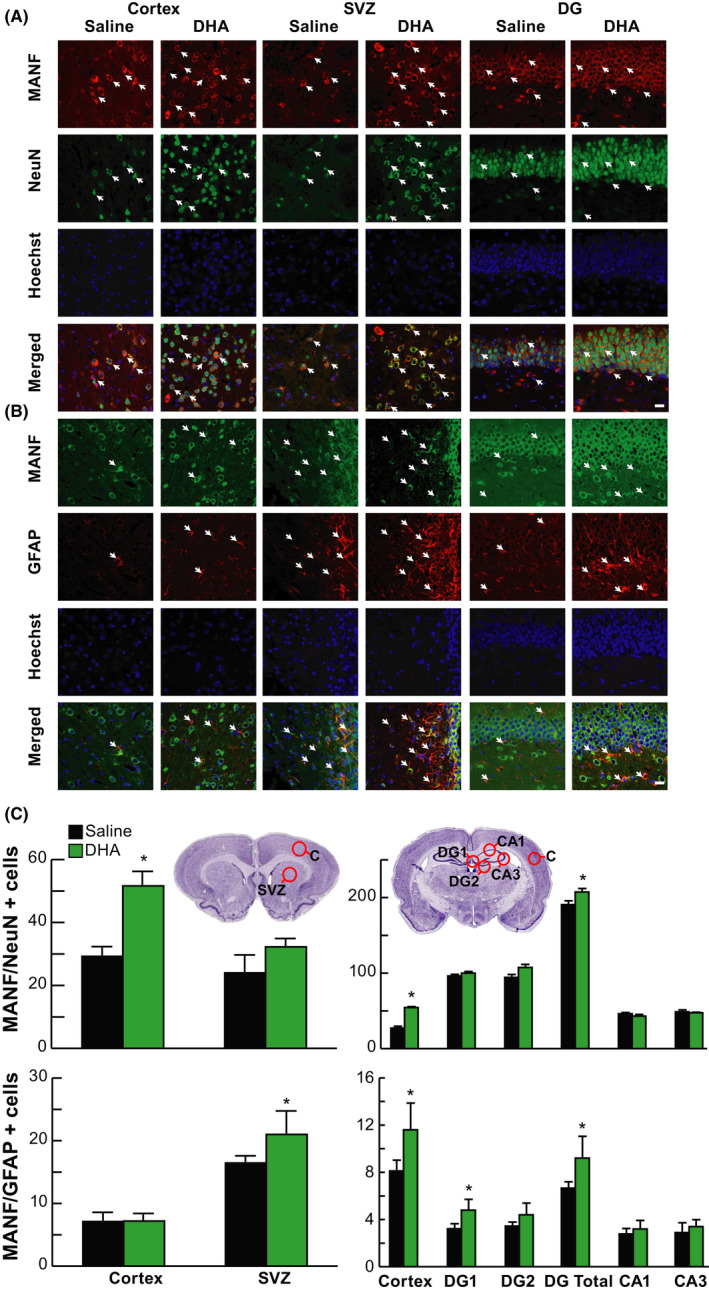
DHA upregulated neuron and astrocyte MANF 24 h after MCAo. Representative images of A, MANF/NeuN^+^ (MANF, red; NeuN, green; Hoechst, blue) and B, MANF/GFAP^+^ (MANF, green; GFAP, red; Hoechst, blue) double immunostaining within cortex, SVZ, and DG. Arrows indicate colocalization of MANF/NeuN (A) and MANF/GFAP (B) positive cells. The images were obtained in the peri‐infarct cortex, SVZ, and DG. Scale bar = 20 µm. C, Location diagrams for cell count and quantification of MANF/NeuN^+^ and MANF/GFAP^+^ cells at +1.2 mm and –3.8 mm of bregma levels. Treatment with DHA increased the number of MANF/NeuN^+^ cells in the cortex and DG and MANF/GFAP^+^ cells in SVZ, cortex, and DG 24h after MCAo. n = 6‐8/group; *P < .05, DHA vs saline; repeated‐measures ANOVA followed by Bonferroni tests. C, peri‐infarct cortex; SVZ, subventricular zone; DG, dentate gyrus; CA1 and CA3, regions of hippocampus

Representative images of MANF/NeuN^+^ and MANF/GFAP^+^ cells at 24 hours after MCAo presented in Figure 2. Treatment with DHA upregulated neuronal (NeuN) and astrocytic (GFAP) MANF expression in the peri‐infarct cortex, SVZ, and DG compared to saline treatment (Figure 2A,B). Double immunofluorescence staining demonstrated that neuronal apoptosis was present in saline‐treated rats, while the administration of DHA significantly promoted neuronal survival (Hoechst) in DG (Figure 2A,B). MANF/NeuN^+^ and MANF/GFAP^+^ cells were counted in the cortex, SVZ, DG, and hippocampus 24 hours after MCAo (Figure 2C). Treatment with DHA increased the quantity of MANF/NeuN^+^ cells in the cortex and DG and MANF/GFAP^+^ cells in SVZ, cortex, and DG 24 hours after MCAo.

### 3.3 DHA improves long‐term survival, attenuates infarct size, downregulates TREM2 expression, upregulates MANF expression, and enhances neurogenesis after stroke

**FIGURE 3:**
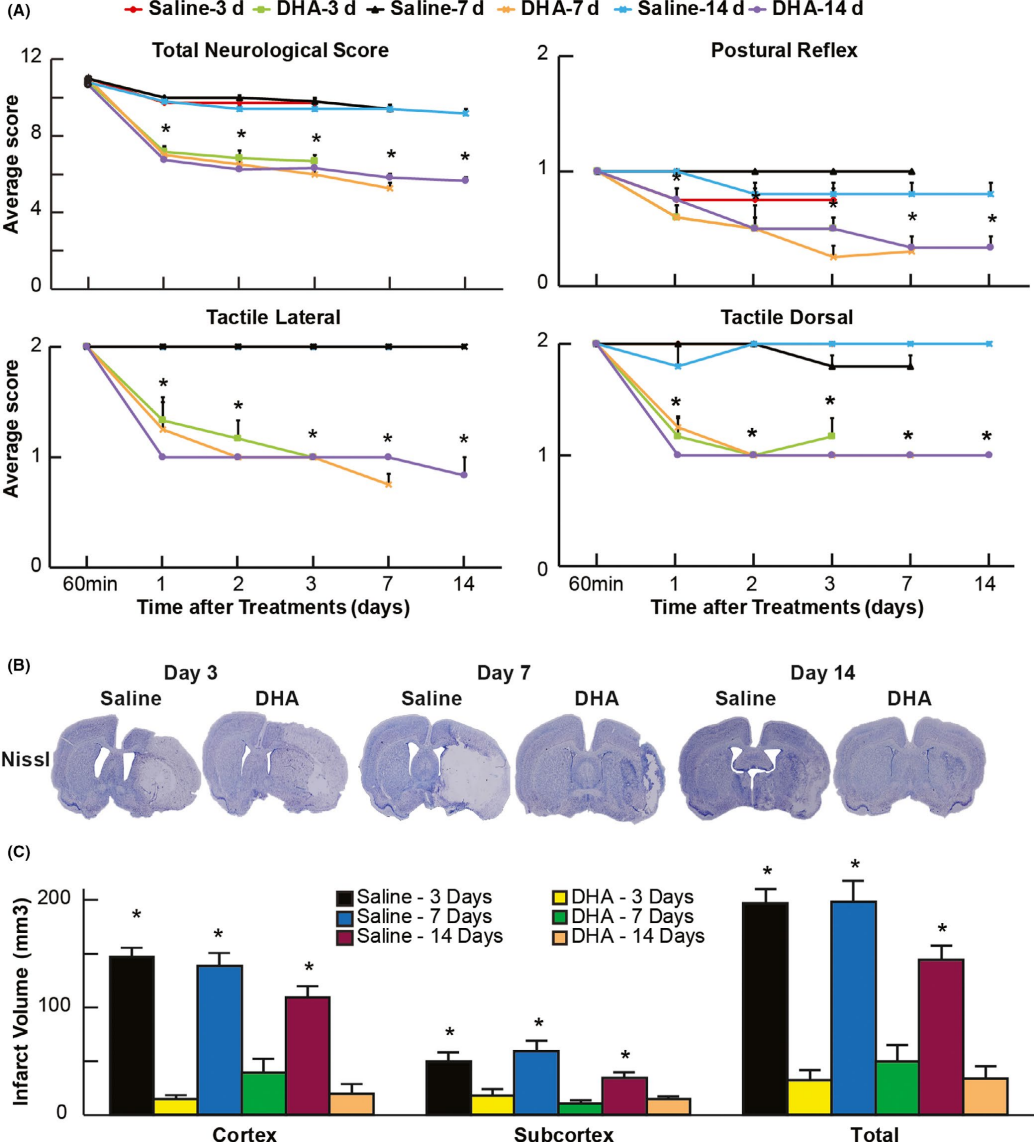
DHA attenuated infarct size and neurological recovery on days 3, 7, and 14 after MCAo. **A**, Neurological scores (normal score = 0, maximal deficit = 12); **B**, cortical, subcortical, and total infarct areas; and **C**, computer‐generated MosaiX‐processed images of Nissl‐stained and ischemia contrast paraffin‐embedded brain sections from all groups. Saline‐treated rats show large cortical and subcortical infarction. In contrast, rats treated with DHA display less damage, mostly in the subcortical area. n = 5‐6/group; *P < .05, DHA vs saline; and repeated‐measures ANOVA followed by Bonferroni tests

Improvement in the total neurological score on days 1, 2, 3, 7, and 14 was observed in rats treated with DHA (Figure 3A) and displayed reduced cortical, subcortical, and total infarct areas at multiple bregma levels (Figure 3B). Saline‐treated rats exhibited consistent pan‐necrotic lesion by Nissl staining, involving cortical and subcortical regions of the right hemisphere, characterized by damage of glial, neuronal, and vascular elements. DHA therapy dramatically reduced infarct size by on days 7 and 14 (Figure 3C).

**FIGURE 4:**
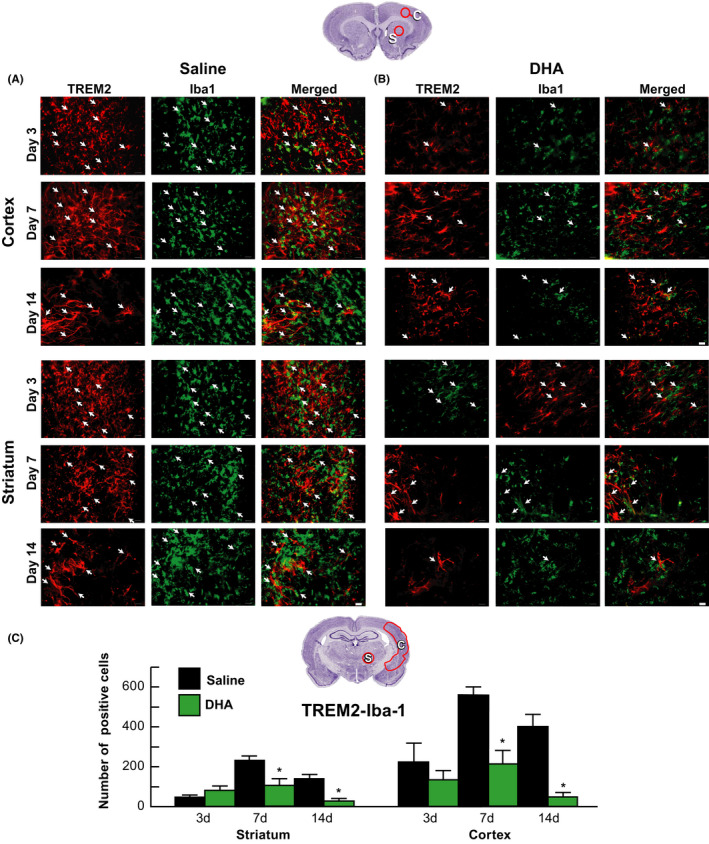
DHA inhibited TREM2 expression in activated microglia after MCAo TREM2 (red) was colocalized with iba1 positive‐activated microglia (green) at cortex and striatum on days 3, 7, and 14 after MCAo. Regional colocalization of TREM2/Iba1 is marked by arrows. Representative images collected from peri‐cortical and striatal infarct areas at bregma level + 1.2 mm are shown. Scale bar = 20 µm

Double immunostaining for TREM2/Iba1 of the peri‐infarct cortex and striatum in saline‐treated rats displays TREM2 expression on activated microglia on Day 7 and was still evident on Day 14 (Figure 4A). On the other hand, DHA inhibited microglial TREM2 expression in the cortex and striatum on days 7 and 14 (Figure 4B,C).

**FIGURE 5:**
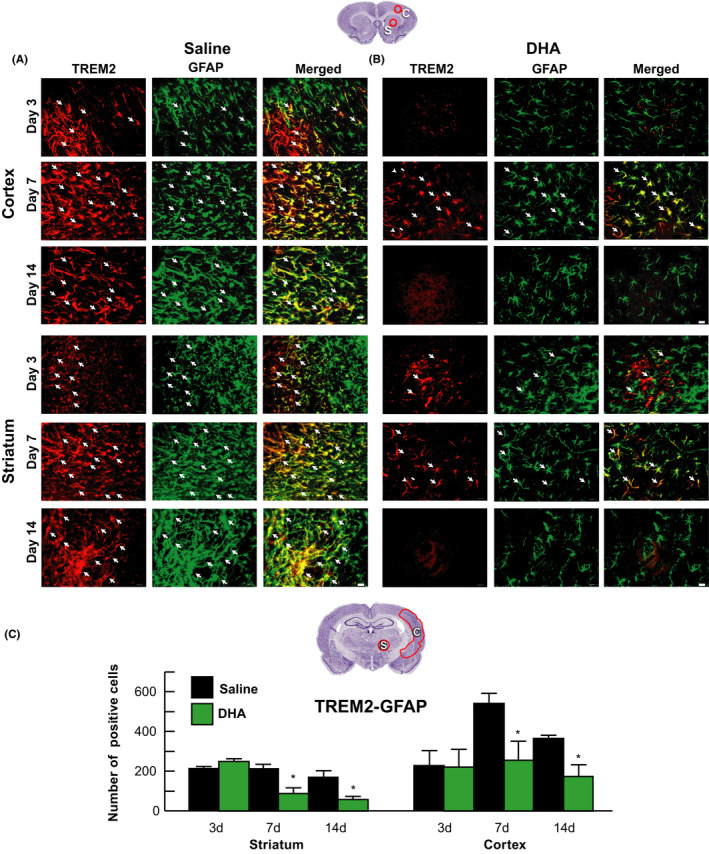
DHA suppressed TREM2 expression in reactive astrocytes after MCAo. Representative images of TREM2^+^ (red) and GFAP^+^ (green) staining in the cortex and striatum on days 3, 7, and 14. Arrows indicate colocalized TRME2/GFAP‐positive cells. Series of images were obtained from peri‐cortical and striatal infarct areas at bregma level + 1.2 mm. Scale bar = 20 µm

Double immunostaining for TREM2/GFAP revealed colocalization in the TREM2‐positive meshwork (Figure 5A,B). Saline‐treated rats exhibited the majority of TREM2‐positive staining in hypertrophic astrocytes (rod shape) over cortical and striatal ischemic border zones on days 3, 7, and 14 (Figure 5A). In contrast, DHA reduced the number of GFAP‐positive astrocytes on days 7 and 14 (Figure 5B). Cellular count for TREM2/GFAP‐positive cells is presented in Figure 5C. DHA decreased TREM2/GFAP on days 7 and 14.

DHA showed high levels of MANF abundance in neurons in the cortex and striatum compared to saline treatment on days 7 and 14. DHA increased MANF‐positive cells in striatum by 90% on Day 7 and by 56% and 45% in peri‐infarct cortex on days 7 and 14.

**FIGURE 6:**
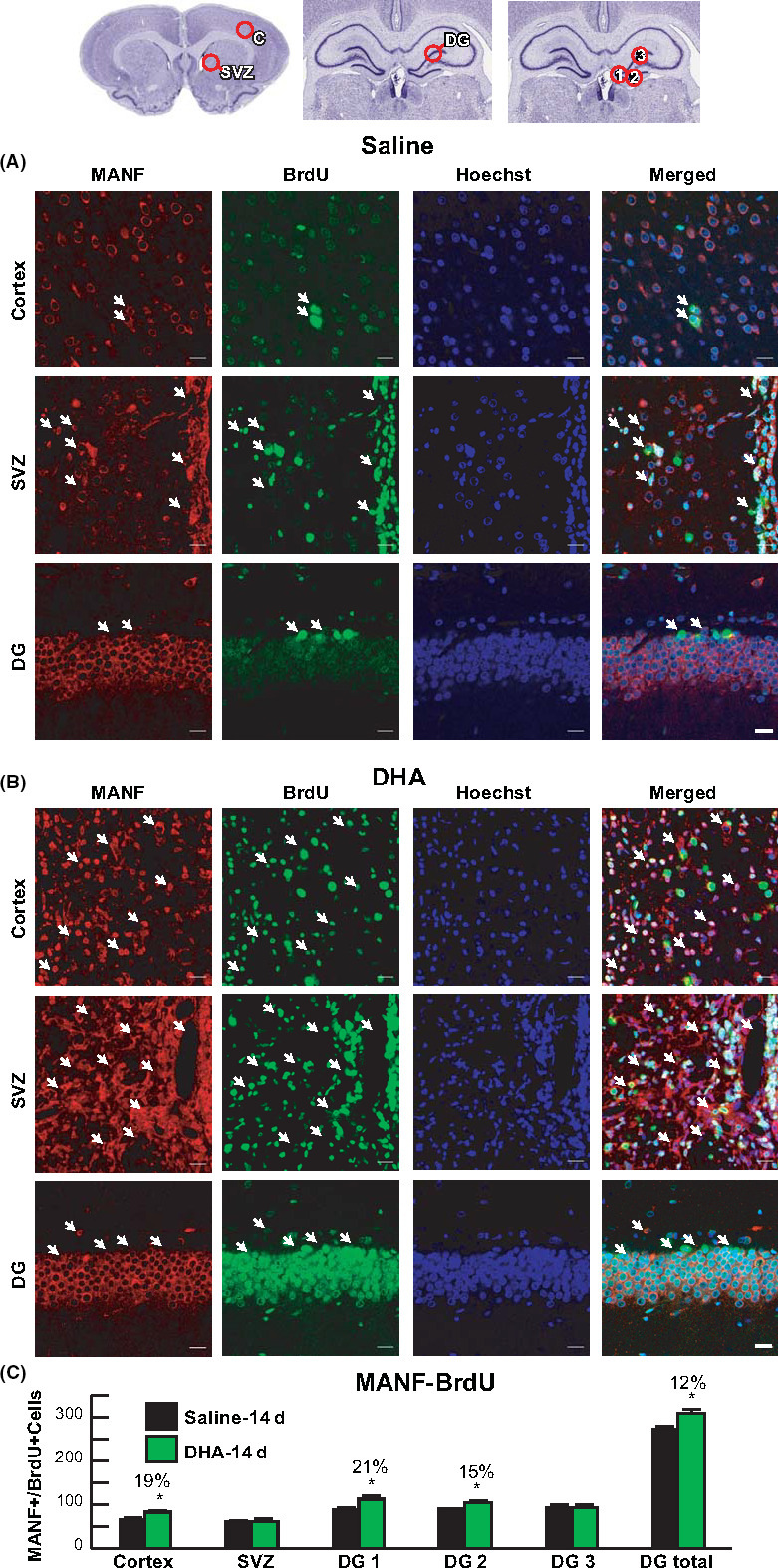
DHA enhances MANF expression and neurogenesis 2 weeks after MCAo. **A**, Diagram of location for the cell count in cortex, SVZ, and three regions of DG (1: DG crest, 2: infrapyramidal DG, 3: suprapyramidal DG). Representative images of MANF^+^ (red), BrdU^+^ (green), and Hoechst^+^ (blue) cells in peri‐infarct cortex, SVZ, and DG from saline (**A**) and (**B**) DHA‐treated rats. Arrows indicate MANF/BrdU double positive cells. Quantification of double‐labeling MANF‐BrdU (**C**). n = 5‐6/group; *P < .05, DHA vs saline; and repeated‐measures ANOVA followed by Bonferroni tests. Images were acquired from cortex and SVZ at bregma level + 1.2mm and DG at bregma level 3.8 mm. Scale bar = 20 µm. Peri‐infarct cortex (C); subventricular zone (SVZ); and dentate gyrus (DG)

To investigate whether DHA enhances neurogenesis after stroke, MANF/BrdU^+^ labeling and Hoechst staining were investigated on Day 14 (Figure 6). DHA increased the number of MANF/BrdU and Hoechst‐positive cells in the cortex, SVZ, and DG compared to saline treatment (Figure 6A,B). Cellular count for MANF/BrdU positive cells is presented in Figure 6C. The number of MANF/BrdU^+^ cells was increased in DHA‐treated rats compared to saline‐treated rats in peri‐infarct cortex and DG on Day 14.

## 4 DISCUSSION

In this study, we examined MANF and TREM2 after MCAo in rats and asked if DHA‐induced neuroprotection engages MANF and TREM2 expression. We observed that neurons and astrocytes expressed MANF and that microglia and astrocytes express TREM2. Moreover, we demonstrated that DHA treatment enhanced MANF, decreased TREM2 expression, reduced ischemic brain injury, activated neurogenesis, and promoted functional recovery after experimental ischemic stroke.

The major source of MANF was not astrocytes since we observed that more neurons than astrocytes express MANF, suggesting that the primary source of MANF is neurons. MANF is stress inducible in glial cells, which are essential for maintaining homeostatic conditions and neuronal signal transmission.^2^ MANF has been shown to inhibit oxygen‐glucose deprivation‐induced cell damage and inflammatory cytokines secretion by astrocytes.^29^ Recombinant human MANF (rhMANF) locally administered to the cerebral cortex before 60 minutes of MCAo reduced ischemic brain injury and promoted behavioral recovery in rats.^5^ Delivery of rhMANF 24 hours after permanent MCAo displays proangiogenic activity, increasing functional cerebral microvessels in the peri‐infarct cerebral cortex.^30^ When MANF administration was delayed by 2 or 3 days after stroke, it promoted functional recovery without affecting lesion volume after distal MCAo.^5^ Intracerebral administration of MANF 2 days following ischemic stroke increased the quantity of phagocytic microglia at 4 days in the peri‐infarct region,^31^, ^32^ and rhMANF promoted neuron proliferation and prevented neuronal apoptosis.^33^

Our study found that MANF was upregulated in neurons and astrocytes on days 1, 7, and 14. DHA improved behavioral deficit and attenuated infarct size; brain swelling on days 7 and 14 increased MANF abundance in the ischemic core, penumbra, and DG. Moreover, DHA augmented neurogenesis 14 days following MCAo, and DHA increased the number of MANF/BrdU and Hoechst‐positive cells in the cortex, SVZ, and DG compared to the saline treatment. The ability of DHA to regulate expression of MANF following ischemia implies a possible mechanism by which DHA is able to protect against further cell and tissue damage.

The microglia cell surface receptor TREM2 is highly expressed in response to neuronal cell damage, and its decreased abundance inhibits prolonged phagocytic microglial activity following injury. However, the significance of this receptor in ischemic stroke remains to be elucidated.^34^ Proinflammatory microglia prevail in the acute phase following stroke, along with enhanced expression of tumor necrosis factor‐alpha (TNF‐α), interleukin (IL)‐1β, IL‐12, and inducible nitric oxide synthase (iNOS). Following ischemic stroke, proinflammatory microglia are necessary to clear neuronal cell debris at the site of injury, though prolonged and increased microglia activation enhances proinflammatory pathways and further exacerbates ischemic damage.^35^, ^36^ Proregenerative microglia secrete antiinflammatory cytokines and remodeling factors, such as vascular endothelial growth factor (VEGF). Nonetheless, proregenerative microglia decline 24 hours following ischemic stroke.^37^ There is uncertainty about whether TREM2 inhibits or promotes microglia activity, along with its effect on inflammatory pathways. One study found that TREM2 is upregulated at the onset of ischemic injury, increasing the activation of microglia at the lesion site, in agreement with reports showing that TREM2 is a proinflammatory mediator and an inhibitor of phagocytic microglial activity.^38^, ^39^ Depletion of microglial TREM2 correlates with exacerbated outcomes from experimental stroke and impeded phagocytosis,^17^ whereas receptor expression led to reduced infarct volumes and neurological recovery.^17^ Additionally, the activation of myeloid cells and phagocytes is diminished in mice without brain TREM2 compared to mice with intact brain TREM2. These results suggest that TREM2 expression participates in poststroke recovery and that resident microglia are essential to recovery.

Our study also established that TREM2 is expressed on macrophages infiltrating tissues from circulation and in astrocytes in the ischemic penumbra and dentate gyrus (DG) on days 7 and 14 after MCAo. Most TREM2‐positive staining was detected in hypertrophic astrocytes (rod shape) over cortical and striatal ischemic border zones on days 3, 7, and 14. DHA inhibited microglial TREM2/Iba1 expression and reduced the number of TREM2/GFAP‐positive astrocytes in ischemic core, penumbra, and DG on days 7 and 14, correlating with improved overall poststroke recovery.

In addition to neuroprotection, translational enabling research strategies currently include the enhancement of poststroke tissue repair using neurorestoration.^40^, ^41^ Neurogenesis activation in the subgranular zone of the DG and the subventricular zone of the lateral ventricles is one mechanism for neurorestoration.^41^ After focal cerebral ischemia, neurogenesis may contribute to functional recovery through the differentiation, proliferation, migration, and integration of newly generated neural cells into circuits.^42^ A candidate to trigger neuro‐reparative mechanisms is DHA,^43^ which promotes neuronal differentiation and neurite growth.^44^ DHA is necessary for nervous system development ^45^ and exhibits antiinflammatory effects, which help to protect brain tissue and promote recovery following ischemic stroke.^20^, ^46^ Previously, we demonstrated that DHA experimental therapy improves behavioral function, promotes cell survival in the ischemic penumbra, decreases infarct volume, and attenuates blood‐brain barrier permeability as well as cerebral edema in rats with a survival time point of one week following focal cerebral ischemia.^20^, ^21^, ^28^, ^47^ DHA also plays a role in memory formation, neurogenesis, and neuroprotection.^22^, ^47^, ^48^ One detrimental consequence of ischemic stroke observed in 70%‐80% of patients that present hemiparesis immediately after stroke, is impaired sensorimotor and cognitive functions.^10^ Our study demonstrates an improvement in overall neurological recovery when DHA is administered starting at 3 hours after onset of stroke, as highlighted by the time‐course of recovery of postural reflex and forelimb placing tests through the 14‐day survival period. In addition, DHA attenuated infarct size and brain swelling on days 7 and 14. We are exploring docosanoids, elovanoids, and related mediators as direct influencers of some of the new bioactivity of DHA described here.^49^

## 5 CONCLUSIONS

We uncover here that MANF and TREM2 abundance is increased after focal cerebral ischemia and that DHA‐potentiated MANF enhanced expression while TREM2 decreased expression. Additionally, DHA triggered enhanced neurogenesis that included augmented MANF/BrdU and Hoechst‐positive cells in the cortex, SVZ, and DG, as well as reduced infarct volume and improved neurological function. Moreover, we suggest that MANF and TREM2 are an attractive target as a therapeutic avenue for ischemic stroke and other cerebrovascular diseases.

## CONFLICTS OF INTEREST

The authors declare that they have no conflict of interest.

## AUTHOR CONTRIBUTIONS

LB and NGB designed the experiments. SHH, RSF, LK, and PKM conducted the experiments. HM, SJM, RBO, and MMR performed image and data analysis. LB and NGB wrote the manuscript. The manuscript was revised and approved by all authors.

## ETHICS APPROVAL

All animal experiments were approved by the Institutional Animal Care and Use Committee of the Louisiana State University Health Sciences Center, New Orleans. All animal procedures were performed to minimize pain or discomfort in accordance with current protocols.

## Supporting information

Fig S1‐S2

## Data Availability

All data generated or analyzed during this study are included in this article.
