# Supplementary figures and images for: DHA modulates MANF and TREM2 abundance, enhances neurogenesis, reduces infarct size, and improves neurological function after experimental ischemic stroke
Source: CNS Neurosci Ther. 2020 Aug 5;26(11):1155–67. doi: 10.1111/cns.13444 (PMC7564189; doi:10.1111/cns.13444)

**Figure S1. Full unedited blots for Figure 1E.**

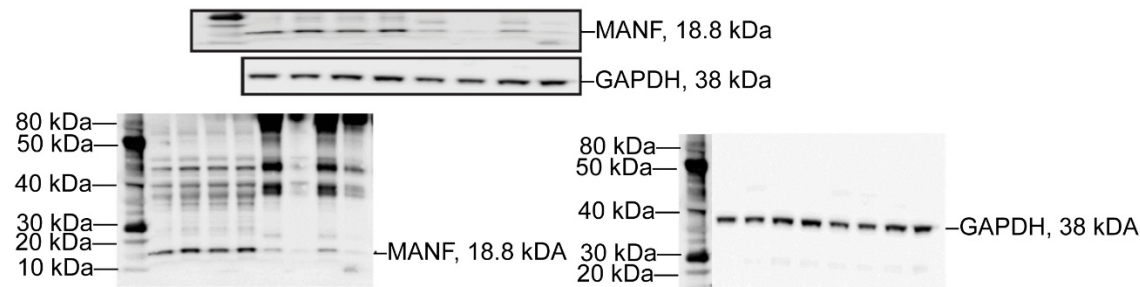

**Figure S2. Full unedited blots for Figure 1F.**

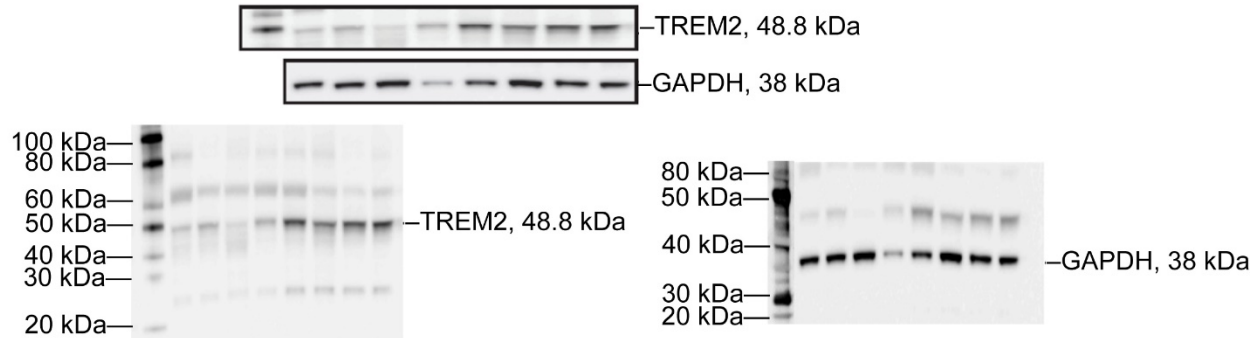

Supplement: Supplementary file 1 — Fig S1‐S2 [file CNS-26-1155-s001.pdf]
